# Supplementary material for: In vitro broad-spectrum antiviral activity of MIT-001, a mitochondria-targeted reactive oxygen species scavenger, against severe acute respiratory syndrome coronavirus 2 and multiple zoonotic viruses
Source: Virus Res. 2024 Feb 5;342:199325. doi: 10.1016/j.virusres.2024.199325 (PMC10851010; doi:10.1016/j.virusres.2024.199325)
Supplement: Supplementary file 1 [file mmc1.docx]

Supplementary information

**Figure S1.**

**(A)**


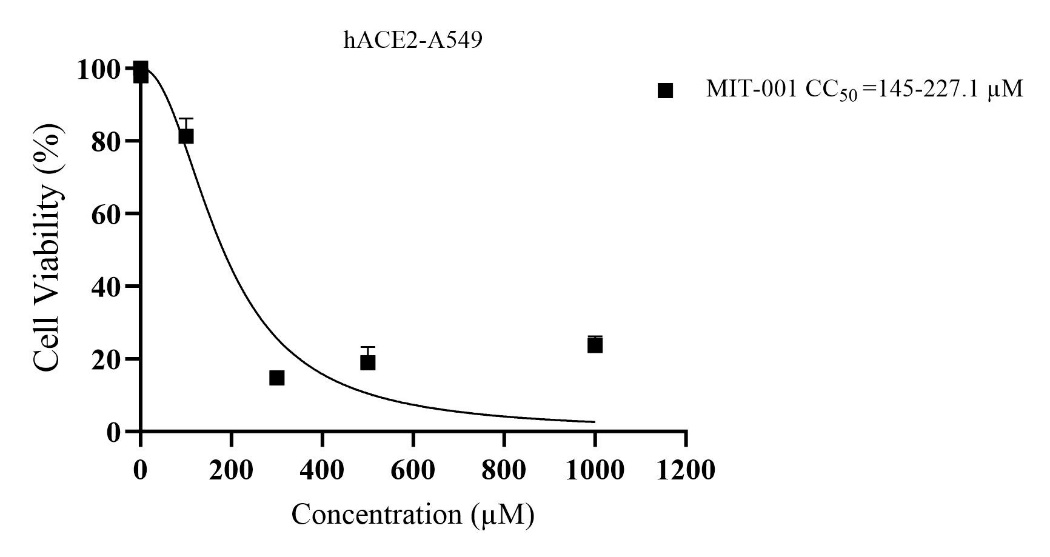


**(B)**

**
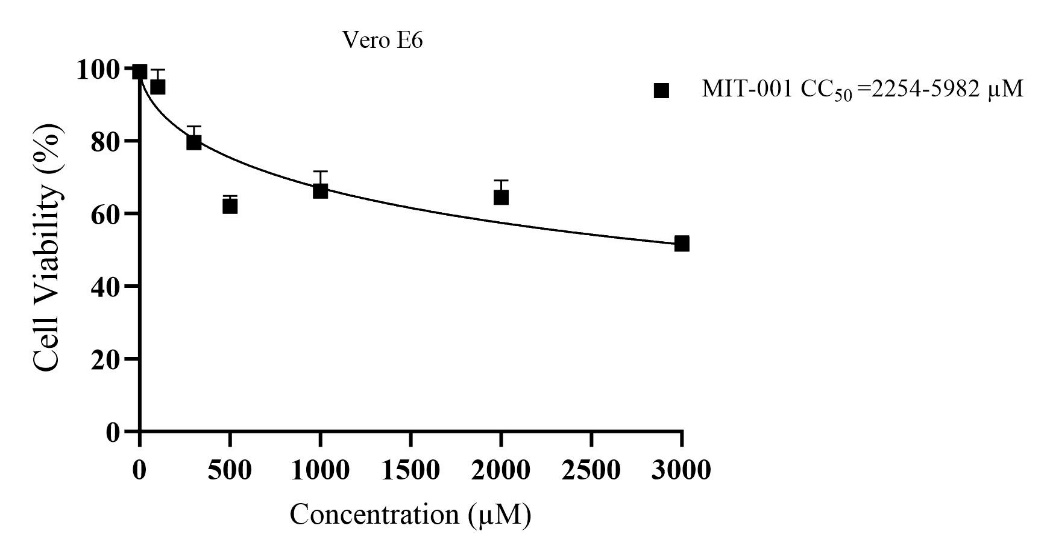
**

**Supplementary Figure S1. Cytotoxicity of MIT-001 in (A) hACE2-A549 and (B) Vero E6 cells.** Cells were treated with DMSO or increasing concentrations of MIT-001 (1 µM-1000 µM in hACE2-A549 and 1 µM-3000 µM in Vero E6 cells) for 48h. At 45h posttreatment, 10µl of CCK-8 solution was added to each well and incubated for 3 hours at 37°C. Fluorescence emission was measured at 450nm. The average fluorescence value of the culture medium background (wells containing no cells) was subtracted from each experimental well. The mean fluorescence emission values of the wells containing DMSO only were considered 100% viability and were used to calculate the percent relative cell viability of the wells containing MIT-001. The percent viability versus the concentration of MIT-001 was plotted using GraphPad Prism v10.1.2, and the values were fitted to a nonlinear regression curve to determine the CC_50_ (the concentration that reduces the total cell number by 50%). The data shown is representative of two independent experiments, where each experiment consisted of triplicate wells per concentration of compound. Results are presented as mean ± SD.

**Supplementary Table 1.**

| **Compound** | **Cell line** | **EC_50_ (μM)** | **SI** |
| --- | --- | --- | --- |
| MIT-001 | Vero E6 | 1.768 | 1274.8-3383.4 |
|  | hACE2-A549 | 1.459 | 99.3-155.6 |

The selectivity index (SI) of MIT-001 (CC_50/_ EC_50)_ against SARS-CoV-2 B.1 in Vero E6 and hACE2-A549 cells.
